# Influence of Jiegeng on Pharmacokinetic Properties of Flavonoids and Saponins in Gancao

**DOI:** 10.3390/molecules22101587

**Published:** 2017-09-21

**Authors:** Yancao Mao, Linxiu Peng, An Kang, Tong Xie, Jianya Xu, Cunsi Shen, Jianjian Ji, Liuqing Di, Hao Wu, Jinjun Shan

**Affiliations:** 1 Jiangsu Key Laboratory of Pediatric Respiratory Disease, Institute of Pediatrics, Nanjing University of Chinese Medicine, Nanjing 210023, China; maoyancao@163.com (Y.M.); sunnyxyl1021@163.com (T.X.); jianyaxu@126.com (J.X.); cunsishen@126.com (C.S.); njustream@163.com (J.J.); 2 Jiangsu Engineering Research Center for Efficient Delivery System of TCM, School of Pharmacy, Nanjing University of Chinese Medicine, Nanjing 210023, China; penglinxiu1016@hotmail.com (L.P.); kanga@njucm.edu.cn (A.K.); diliuqing@hotmail.com (L.D.)

**Keywords:** *Radix platycodonis*, *Glycyrrhiza uralensis Fisch*, pharmacokinetics, absorption, metabolism

## Abstract

Jiegeng Gancao decoction, which is composed of Jiegeng and Gancao at a weight ratio of 1:2, was widely used for treating pharyngalgia and cough for thousands of years. Our previous work indicated that Gancao could increase the systemic exposure of platycodin D and deapio-platycodin D, two main components in Jiegeng. However, whether Jiegeng could alter the pharmacokinetics of the main compounds in Gancao is still unknown. Thus, the purpose of this study was to compare the oral pharmacokinetics of flavonoids and saponins from Gancao alone vs. after co-administration with Jiegeng. Furthermore, Caco-2 cell transport and fecal hydrolysis were investigated to explain the altered pharmacokinetic properties. Pharmacokinetics results suggested that the bioavailability of liquiritin, isoliquiritin, glycyrrhizin and its metabolite, glycyrrhetinic acid, could be improved while bioavailability of liquiritigenin and isoliquiritigenin deteriorated when co-administered with Jiegeng. The Caco-2 transport study showed no significant difference of the P_app_ values of the main components in Jiegeng Gancao decoction when compared with those in Gancao decoction (*p* > 0.05). The in vitro metabolism study suggested that saponins and flavonoids glycosides in Gancao were influenced and the metabolic characteristics of most ingredients were consistent with pharmacokinetic results, such as liquiritin and glycyrrhetinic acid. The hydrolysis of liquiritigenin and glycyrrhizin observed with fecal lysate in vitro appeared consistent with the oral pharmacokinetics. Based on experiments, the pharmacokinetic profiles of six components in Gancao were influenced by Jiegeng. The metabolic process might partially contribute to the altered pharmacokinetic behavior. The metabolism of some components of Gancao appeared to be inhibited when coadministered with Jiegeng, possibly by the Jiegeng constituent platycodin.

## 1. Introduction

**Figure 1:**
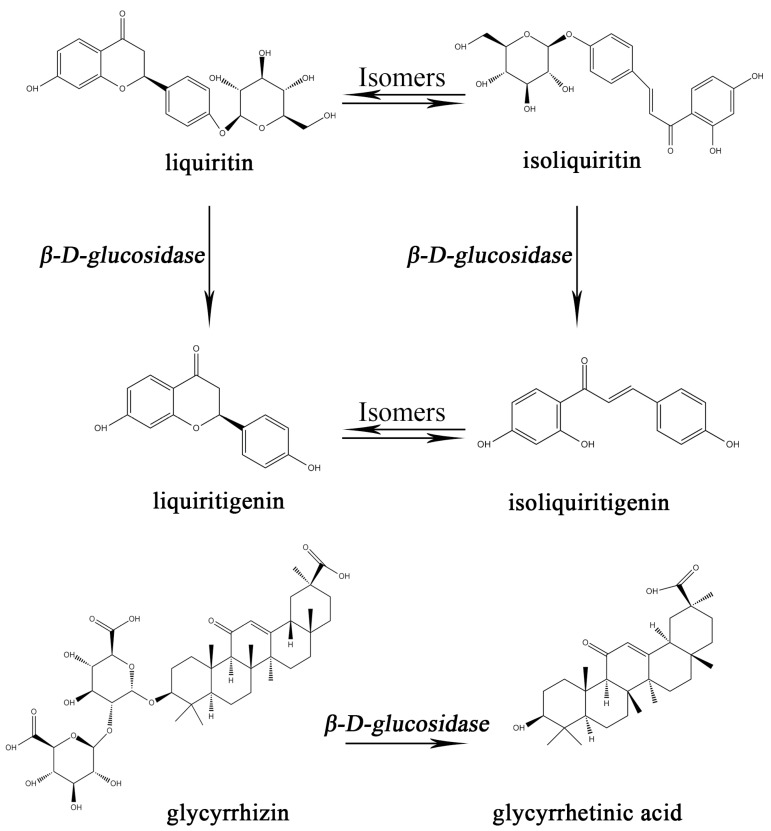
The chemical structures of six components in Gancao and the relationship of between them are annotated in the figure as appropriate.

Licorice, also called Gancao (GC) in China, was derived from the dried roots and rhizomes of the Glycyrrhiza species. It was widely used for flavoring additives and confectionery in the food industry due to its sweet taste in most parts of the world [1,2]. Additionally, Gancao was clinically used for treating wounds, diabetes, cough, and tuberculosis in China, Japan, Korea, and India [3,4,5,6]. As the most frequently used herbal medicine worldwide, Gancao appears in about 60% of traditional Chinese medicine (TCM) prescriptions [7,8,9] and confectionery products [10]. To date, more than 400 compounds have been isolated from Gancao, of which the main constituents being saponins and flavonoids [11], which are believed to be useful in pharmacology. Among these compounds, liquiritin (LQ), isoliquiritin (ILQ), liquiritigenin (LG), isoliquiritigenin (ILG) and glycyrrhizin (GLY) gained great attention for their validated anti-inflammatory and anti-virus effects [12,13]. The chemical structure of each compound is shown in Figure 1.

*Platycodon grandiflorus*, commonly named Jiegeng (JG) in China, was the root of *Platycodon grandiflorum A.DC* (*Campanulaceae* family). Traditionally, it has been consumed as food, as well as a folk remedy for ameliorating bronchitis, asthma, pulmonary tuberculosis, and inflammatory conditions [14,15,16]. To date, more than 40 triterpenoid saponins, have been reported to existing in Jiegeng [14,17,18]. Gancao was usually used together with Jiegeng to treat pharyngitis, cough, etc. [19]. Gancao and Jiegeng, at a ratio of 2:1, was first described as Jiegeng Gancao Decoction or Jiegeng Tang (JG–GC) in the medical classic ‘Shang-han-lun’ to treat abscess of lung based on its demonstrated capacity to act as an expectorant, resolving phlegm and improving air flow to the lungs [20]. It was reported that these two plants had a distinct synergistic effect working as an anti-inflammatory and eliminating phlegm [21].

Several studies have investigated the pharmacokinetics of the flavonoids and saponins of Gancao [22,23]. Based on these studies, the pharmacokinetic profiles of the main active components of Gancao, liquiritin, liquiritigenin and glycyrrhizic acid, were systematically determined. However, the effects of Jiegeng on the pharmacokinetics of the active components in Gancao were still unclear.

In this study, the influence of Jiegeng on the pharmacokinetics of five active constituents—liquiritin, isoliquiritin, liquiritigenin, isoliquiritigenin and glycyrrhizin and one metabolite of glycyrrhizin, glycyrrhetinic acid (GA)—in Gancao were evaluated. Furthermore, Caco-2 cell transport and fecal hydrolysis experiments were employed to investigate the absorptive and metabolic behavior of the six active components mentioned above in Gancao alone and in Jiegeng Gancao decoction.

## 2. Results and Discussion

### 2.1. Oral Pharmacokinetics Study

**Figure 2:**
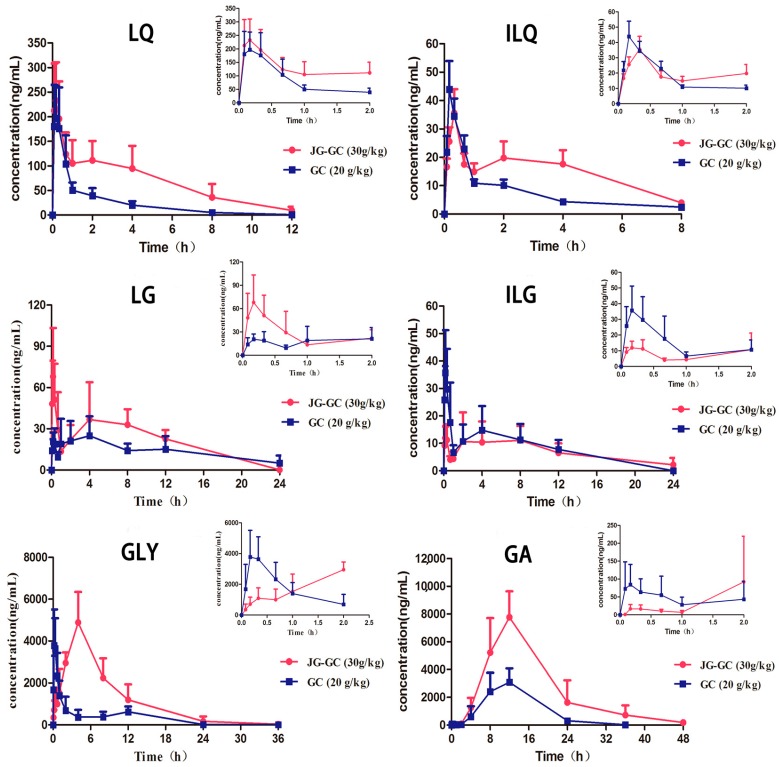
Time–concentration profiles of LQ, ILQ, LG, ILG, GLY and GA in rat plasma from Group 1 (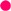): JG–GC (30 g/kg, p.o.); Group 2 (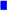): GC (20 g/kg, p.o.). The upper right corner of each drug curve shows the corresponding drug profile within 2 h.

The pharmacokinetic parameters and mean plasma concentration–time profiles were presented in Table 1 and Figure 2.

Among the four flavonoids, we noticed that the AUC_(0–t)_ (AUC: area under the curve) of LQ and ILQ, two flavonoid glycosides, increased in the presence of Jiegeng in different ways. The AUC_(0–t)_ of LQ increased about 3.6 times (*p* < 0.05), while the value of ILQ increased about 1.8 times (*p* > 0.05). In contrast, the AUC_(0–t)_ of LG and ILG, two flavonoid aglycones, decreased, with the AUC_(0–t)_ of LG decreasing by 42.9% (*p* < 0.01), while the value AUC_(0–t)_ decreased by 40.3% (*p* < 0.05) for ILG when co-administered with Jiegeng. In a similar manner to the flavonoid glycosides, the AUC _(0–t)_ of GLY and its metabolite GA also increased significantly, with the AUC_(0–t)_ of GLY and GA increasing about 3.0 times (*p* < 0.05) and 2.9 times (*p* < 0.05), respectively. These results indicate that Jiegeng might significantly improve the bioavailability of glycosides, including flavonoids (LQ and ILQ) and saponins (GLY and GA), while Jiegeng may decrease the bioavailability of LG and ILG in Gancao.

Another obvious difference could be observed from the time–concentration profiles of GLY and GA. The T_max_ of GLY was significantly prolonged by 12.38 times in JG–GC (*p* < 0.05), meanwhile, the C_max_ of GA markedly increased about 2.5 times (*p* < 0.05) when combined with Jiegeng. This suggests that more GLY was metabolized into GA under the influence of Jiegeng during this prolonged period. In summary, the AUC of LQ, ILQ, GLY and GA in the JG–GC group increased by 3.6, 1.8, 3.0, and 2.9-fold, respectively, while the AUC of LG and ILG decreased compared to the GC group. Previous work revealed that Gancao can increase the AUC of platycodin D by six times [24], in this study, more evidence has been added indicating that main components in the two herbs may interact after oral administration. To determine the alterations to the pharmacokinetic characteristics between the GC group and the JG–GC groups, two in vitro drug absorption and metabolism models were applied to investigate the potential herb–herb interaction.

### 2.2. Transcellular Transport of LQ, LG and GLY across Caco-2 Cell Monolayer

The cytotoxicity of the JG–GC on the Caco-2 cells was determined through a MTT test (a method of detecting cell survival and growth) prior to carrying out the transport experiments. The concentration and corresponding cell viability was calculated for each substance to determine the optimum concentration—those not exhibiting cytotoxicity where the living rate of cells above 80 percent—to use in transportation assays.

**Figure 3:**
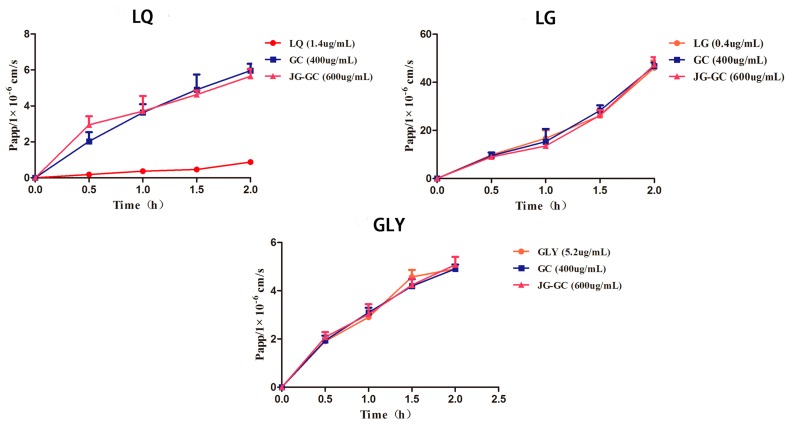
P_app_ profiles for the analytes in different groups (mean ± SD, n = 3).

According to P_app_ for the analytes, as shown in Table 2 and Figure 3, LG exhibited good permeability (P_app_ > 10^−5^ cm/s), suggesting that LG could be easily absorbed. Meanwhile, the P_app_ values of LG and GLY increased with the extension of time in each group. However, obvious differences did not exist between LG alone and LG combined with GC or JG–GC. This result demonstrated that there were no other ingredients in Jiegeng or in Gancao that affected the absorption of GLY and LG. Conversely, the P_app_ value of LQ, a flavonoid glycoside in Gancao, was less than 10^−6^ cm/s in the LQ without GC or JG–GC but increased in the GC and JG–GC groups, however, no difference appeared between these two groups. The results mentioned above suggest that LQ has poor absorption and some ingredients in Gancao could promote the absorption of LQ. 

The absorptions of the main active ingredients in Gancao were not significantly changed after co-administration with Jiegeng, indicating that pharmacokinetic characteristic changes were not a result of drug absorption.

### 2.3. Hydrolysis of LQ, LG and GLY in Fecal Lysates

**Figure 4:**
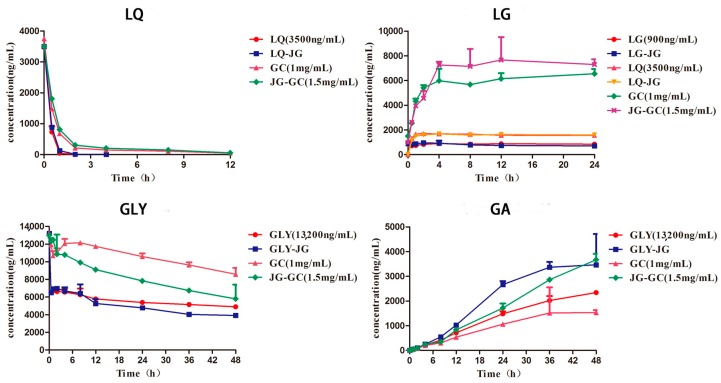
Hydrolysis of Gancao constituents in rat fecal lysates (mean ± SD; n = 3).

Observations of the metabolic profiles (Figure 4) of LQ in the four groups indicate that LQ could be metabolized, for the most part within 2 h. However, the metabolic rate increased slightly in the LQ or LQ–JG group. These phenomena suggest that some ingredients in Gancao inhibited the hydrolysis of LQ. LG, the aglycone of LQ, could not be hydrolyzed in the fecal lysates, but converted from LQ, with the aid of intestinal flora, as previous research reported [25]. This conclusion was validated through the growth trend profiles of LG in six different groups. As shown in Figure 3, the concentration of LG was stable after 2 h in each group, with the exception of the GC and JG–GC groups. Meanwhile, the concentration of LG increased in varying degrees between the GC and JG–GC groups, indicating that some other components could be hydrolyzed to LG, except LQ. For example, isoliquiritin and isoliquiritigenin, two main flavonoids in Gancao, could be converted to LG via metabolism and isomerization [26].

About 50% GLY was metabolized into GA as GLY alone or co-administered with Jiegeng within 0.5 h in the fecal lysates. In contrast, the metabolic rate of GLY decreased in both the GC and JG–GC groups. Furthermore, the concentration of GLY in the JG–GC group decreased rapidly in comparison to the GC group. These results could be verified using the profiles of GA in each group, with many reports demonstrating that GA is the secondary metabolite of GLY by intestinal flora [27]. As our previous research reported, the hydrolysis of platycodin D is inhibited in JG–GC group [24], and it deserves further experimentation to explore this interaction.

In summary, the metabolism of some active components in Gancao has changed in rat fecal lysates after co-administration with Jiegeng. β-d-Glucosidase existing in fecal could hydrolyze the saponins and flavone glycosides, inhibiting or promoting the metabolism of some substances [28], with the action of other ingredients in the drug. This might be an important factor responsible for the changed pharmacokinetic profiles of the active ingredients in JG–GC.

## 3. Materials and Methods 

### 3.1. Reagents and Materials

Reference compounds of liquiritin (LQ), isoliquiritin (ILQ), liquiritigenin (LG), isoliquiritigenin (ILG), glycyrrhizin (GLY), glycyrrhetinic acid (GA) and hesperidin (internal standard, IS) were purchased from National Institute for the Control of Pharmaceutical and Biological Products (Beijing, China). The purity of each compound was more than 98% (HPLC). The two plant materials of Gancao and Jiegeng were purchased from Nanjing Yifeng Pharmacy (Nanjing, China) and were authenticated by Dr. Shengjin Liu (Department of Pharmacy, Nanjing University of Chinese Medicine, Nanjing, China).

HPLC-grade acetonitrile was purchased from Merck (Darmstadt, Germany), and formic acid with a purity of 99% was of HPLC grade (ROE, Newark, NJ, USA). Deionized water (18 MΩ) was prepared by a Milli-Q system (Millipore, Milford, MA, USA) for UHPLC analysis and all solutions or dilutions. Other reagents and chemicals were of analytical grade. Dulbecco’s modified Eagle’s medium (DMEM), fetal bovine serum (FBS), 0.1% EDTA-1.25% trypsin (EDTA/trypsin), penicillin-streptomycin, nonessential Amino Acids and Hank’s Balanced Salt Solution without Calcium & Magnesium (HBSS) were all obtained from Gibco Laboratory (Invitrogen Co, Grand Island, NY, USA).

### 3.2. Preparation of Sample Extracts

Jiegeng Gancao decoction (JG–GC) was composed of Jiegeng (100 g) and Gancao (200 g), and the plants were immersed in 13-fold deionized water for 0.5 h and then decocted to boil for 2 h. The extracted solution was filtered through two layers of gauze. Ethanol was added to precipitate the polysaccharide and protein. This sample was kept for 24 h at 4°C and then filtered through the filter paper. Finally, the filtrate was concentrated to obtain JG–GC with a final concentration of 3.0 g/mL (equivalent to dry weight of raw materials). Gancao decoction (GC, 200 g) was prepared as the above identical procedure, and the final concentration was 2.0 g/mL. All the extracts were kept in −70 °C until use.

### 3.3. Animals

Male Sprague-Dawley rats (weighing 200 ± 20 g) were supplied by the Animal Core Facility of Nanjing Medical University (Certificate No. SCXK-2008-0033) and kept in the laboratory condition (temperature, 22 ± 2 °C; relative humidity, 45~60%). The animals were acclimatized to the facilities for 3 days, and then fasted for 12 h but allowed free access to water prior to experiment. The animal experiments were conducted in accordance with protocols approved by the Animal Ethic Committee of Nanjing University of Chinese Medicine.

### 3.4. Pharmacokinetic Study

The rats were randomly divided into two experimental groups (n = 6 per group), including JG–GC group and GC group. Rats were administrated with JG–GC or GC at oral dose at 30 g/kg and 20 g/kg. The concentration of LQ, ILQ, LG, ILG and GLY in JG–GC and GC were determined using our validated UHPLC method, and the contents of these compounds in JG–GC and GC were shown in Table 3. About 250 µL blood samples were collected into heparinized tubes via the postorbital venous plexus veins at 0.083, 0.167, 0.33, 0.67, 1, 2, 4, 8, 12, 24, 36 and 48 h after drug administration.

The samples were immediately centrifuged at 5000 rpm for 6 min, and 100 µL of plasma was spiked with 10 µL IS solution (92 ng/mL) by vortexing for 2 min. All analytes could be extracted from plasma with one-step precipitation by adding 300 µL methanol and vortexing for 5 min. The well-vortexed solutions were centrifuged at 17,000 rpm for 10 min. Finally, 5 µL of the supernatant was injected into the UHPLC-MS/MS system for analysis.

To obtain the pharmacokinetic parameters of the six analytes, the time-concentration data was processed by non-compartmental analysis (DAS 3.2.6 software, Chinese Pharmacological Association, Beijing, China). The pharmacokinetic parameters, including T_max_, Cmax, t_1/2_, AUC _(0–t)_, and AUC_(0–∞)_ of the four flavonoids (LG, ILG, LQ, ILQ) and two saponins (GLY, GA) in both groups were calculated and compared. The data was presented as mean ± SD for each group, if not specified otherwise. Significance is assessed by Student’s t-test, with *p* < 0.05 considered to be statistically significant.

### 3.5. Cell Culture

The human colon adenocarcinoma cell line, Caco-2, cells were obtained from the Shanghai Institute of Biochemistry and Cell Biology (Shanghai, China). Cell passages from the 30th to 60th generations were used in the experiment. Cells were maintained at 37 °C in an atmosphere of 5% CO_2_ and 90% relative humidity, growing routinely on 75 cm^2^ plastic culture flasks (Becton Dickinson, Franklin Lakes, NJ, USA) in DMEM, with 10% (*v*/*v*) FBS, 1% (*v*/*v*) non-essential amino acid solution, and 1% (*v*/*v*) penicillin–streptomycin. The medium was replaced every 2–3 days after incubation. The cells were seeded at a density of 5 × 104 cells/cm^2^ onto a permeable polycarbonate insert (0.6 cm^2^, 0.45 μm pore size, Millipore, Billerica, MA, USA) in 24-well tissue culture plates (NUNC, Roskilde, Denmark) and grown for 21 days before the transporter experiment. The resistances of monolayers were assessed using Millcell ERS-2 transmembrane resistance meter (Millipore, Billerica, MA, USA) on days 3, 5, 10, 14, 18 and 21. The transepithelial electrical resistance (TEER) values were calculated to examine its integrity. Only the Millicell membranes with monolayer cells that met the defined criteria with TEER values above 300 Ω·cm^2^ were used for transport studies.

### 3.6. Transport of LQ, LG and GLY across Caco-2 Cell Monolayer

The Caco-2 cell monolayers were washed three times with HBSS (pH 7.4), and placed in a shaking incubator for 30 min to remove impurities. The transport study was conducted by adding 0.4 mL of LQ solution (1.4 µg/mL), LG solution (0.4 µg/mL), GLY solution (5.2 μg/mL), GC solution and JG–GC solution. The concentration of each analyte in the GC and JG–GC solutions was measured to be equal to the targeted analyte. Furthermore, the difference was statistically negligible. These solutions were applied to the apical side of the cell monolayer, with 0.6 mL of blank HBSS applied to the basolateral side. Aliquots of 0.1 mL and 0.15 mL of samples were taken sequentially from the donor and receiver sides, respectively, after shaking at 50 rpm for 0, 30, 60, 90, 120 min. Drug solutions and HBSS medium were added to the apical or basolateral side immediately to compensate for volume lost due to sampling. A 10 μL internal standard (92 ng/mL of hesperidin in acetonitrile) was added to 100 µL of sample, and the mixture was vortexed for 5 min. All samples were nitrogen-dried and reconstituted with 20 μL methanol for UHPLC-MS/MS analysis.

The apparent unidirectional permeability, from apical to basolateral side (P_app_(a-b)), was calculated according to the following equation: P_app_= [(dQ/dt)]/[A × C_0_], where the dQ/dt (µg/min) was the drug permeation rate, A was the cross sectional area (0.6 cm^2^), and C_0_ (µg/mL) was the initial concentration of each drug solution in the donor compartment at t = 0 min.

### 3.7. Fecal Lysates Preparation

Fresh feces were collected from ten male normal SD rats, of which about 1 g was immediately mixed with 9 mL ice-cold PBS (pH 7.4), and vortexed for 5 s followed by filtering through four layers of gauze. For preparing fecal lysates, the filtrate was centrifuged at 1000 rpm for 15 min. The pellet was re-dissolved in 10 mL ice-cold PBS, and the mixture was sonicated in an ice water bath for 45 min, followed by centrifugation at 15,000 rpm at 4 °C for 30 min. All supernatant was stored at −70 °C.

### 3.8. Metabolism of LQ, LG and GLY in Fecal Lysates

Thawed fecal lysates (100 µL) were transferred to disposable glass vials, and then 10 µL of each drug solution with 90 µL PBS was added to obtain a 200 µL incubation volume. The concentrations of the analytes in the drug solutions are listed in Table 4. The mixture was shaken at 37 °C, and samples of 100 µL were collected at 0.5, 1, 2, 4, 8, 12, 24, 36 and 48 h, after which 10 µL of 92 ng/mL hesperidin was added. The mixture was vortexed for 2 min and centrifuged at 17,000 rpm for 10 min. The supernatant was injected into the UHPLC/MS-MS to determine the remaining concentration of each analyte. The experiments were carried out in triplicate.

### 3.9. Quantification of Flavonoids and Saponins in Biological Matrices 

The UHPLC-MS/MS was an ultimate 3000 series UHPLC system (Thermo Fisher, San Jose, CA, USA), consisting of a quaternary pump, an autosampler, a vacuum degasser, and a statically controlled column apartment equipped with a Thermo scientific quantum vantage™ mass spectrometer system (Thermo Fisher, San Jose, CA, USA), which coupled with an electrospray Ionization (ESI) source. All data was analyzed by Xcalibur Software from Thermo-Fisher Scientific. Flavonoids and saponins in Gancao and hesperidin were monitored in the negative mode (ESI−). Some significant mass spectrometry parameters, such as spray voltage, shealth gas pressure, aux gas pressure, vaporizer temperature and capillary temperature were set at 3.0 kV, 45 arb, 25 arb, 450 °C and 350 °C, respectively. The Q1 and Q3 quadrupoles were set at unit resolution with different collision energy (CE) and S-lens. The quantification was performed by the selected reaction monitoring (SRM) of the transitions of *m*/*z* 254.964–134.939 for LG/ILG (CE of 18 eV; S-lens at 64 V), 416.996–254.947 for LQ/ILQ (CE of 22 eV; S-lens at 93 V), 821.134–350.827 for GLY (CE of 39 eV; S-lens at 160 V), 469.120–425.289 for GA (CE of 37 eV; S-lens at 160 V) and *m*/*z* 609.200–301.100 for hesperidin (CE of 27 eV; S-lens at 129 V), respectively. The chromatographic analysis was achieved on a Hypersil Gold C18 column (100 mm × 2.1 mm, 1.9 µm particle size; Thermo, San Jose, CA, USA). The mobile phase consisted of 0.05% formic acid in water (A) and acetonitrile (B) at a flow rate of 0.4 mL/min. The UHPLC gradient system began with 15% B at 0–1 min, 15–70% B at 1–6 min, and 70–100% B at 6–7 min. The column temperature was maintained at 40 °C, while the sample-tray temperature was kept at 4 °C.

The UHPLC-MS/MS analysis of LQ, ILQ, LG, ILG, GLY and GA in rat plasma, HBSS buffers and fecal lysates have been studied [24] and the methods have been validated, respectively. Other substances did not interfere with the detection of these components in each biological sample. The intra- and inter-batch precision and accuracy were < 20% for all quality control samples. In addition, all the substances tested in each biological matrix were stable, and the linear range and LLOQ of each analyte in different samples are shown in Table 5.

## 4. Conclusions

The in vivo process of drug research plays a major role in exploring the material basis of traditional Chinese medicine. This study investigated the in vivo and in vitro interaction of the active ingredients in Jiegeng Gancao decoction systematically from the aspects of pharmacokinetics, absorption and metabolism, elucidating the integral mechanism for drug combination. The result of the Caco-2 cell transport experiment showed that the P_app_ of the main active ingredients were the same between Gancao alone and Jiegeng Gancao decoction, which indicated that the absorption of the active ingredients was not affected after combination with Jiegeng. However, microbial metabolism of Gancao saponin and flavonoid glycosides were inevitably influenced when co-administrated with Jiegeng. Consequently, the altered pharmacokinetics between the GC and JG–GC groups may be attributable to microbial metabolism, rather than intestinal absorption. In summary, these results provided in vivo evidence of the efficacy of this herbal combination and its potential for clinical application.

## Figures and Tables

**Table 1:** Pharmacokinetic parameters of the analytes in rat plasma after oral administration of Gancao (GC) and Jiegeng (JG)–GC (mean ± SD, n = 6).

**Parameter**	**LQ**		**ILQ**		**LG**	
	**GC**	**JG–GC**	**GC**	**JG–GC**	**GC**	**JG–GC**
*C*_max_ (ng/mL)	2.48 ± 72.6	294 ± 52.4	45.9 ± 14.5	35.6 ± 7.08	47.9 ± 9.24	37 ± 9.79
t_1/2_ (h)	2.02 ± 0.90	2.54 ± 0.81	4.78 ± 0.84	2.93 ± 0.89	2.80 ± 0.19	2.54 ± 0.81
T_max_ (h)	0.14 ± 0.043	0.13 ± 0.046	0.25 ± 0.09	0.33 ± 0	0.78 ± 0.05	1.29 ± 0.05
AUC_(0–t)_ (ng/mL·h)	301.9 ± 58.1	1104.5 ± 270 *	75.3 ± 17.6	136 ± 36.5	533.3 ± 191.8	304.4 ± 59.1 **
AUC_(0–∞)_ (ng/mL·h)	303 ± 58.4	1106 ± 269 *	76.6 ± 17.2	137 ± 36.5	545 ± 213	262 ± 55.6 **
**Parameter**	**ILG**		**GLY**		**GA**	
	**GC**	**JG–GC**	**GC**	**JG–GC**	**GC**	**JG–GC**
*C*_max_ (ng/mL)	45.2 ± 13.3	17.3 ± 5.09 *	4102 ± 1307	5190 ± 1228	3109 ± 1007	7848 ± 1862 *
t_1/2_ (h)	3.37 ± 0.33	4.41 ± 1.04	3.03 ± 0.48	3.43 ± 0.87	4.22 ± 0.87	6.00 ± 1.78
T_max_ (h)	1.56 ± 0.25	1.78 ± 0.096	0.39 ± 0.11	4.83 ± 0.89 *	11.3 ± 1.63	11.3 ± 1.63
AUC_(0–t)_ (ng/mL·h)	201.8 ± 54.4	120.4 ± 31.5 *	13,530.7 ± 4038	41,241.1 ± 10,290 *	40,353.5 ± 9018.2	117,529.5 ± 20,640.3 *
AUC_(0–∞)_ (ng/mL·h)	205 ± 52.9	129 ± 21.5	13,532 ± 4037	41,248 ± 10,284 *	40,418 ± 8993	118,998 ± 21,515 *

*: JG–GC vs. GC, * < 0.05, ** < 0.01.

**Table 2:** The P_app_ values for the analytes in different groups (mean ± SD, n = 3).

Time (min)	P_app_-LQ (1 × 10^−6^ cm/s)			P_app_-LG (1 × 10^−6^ cm/s)			P_app_-GLY (1 × 10^−6^ cm/s)		
	LQ	GC	JG–GC	LG	GC	JG–GC	GLY	GC	JG–GC
30	0.18 ± 0.01	2.03 ± 0.51 **	2.95 ± 0.47 ^##^	9.80 ± 0.78	9.48 ± 1.35	8.98 ± 0.70	1.91 ± 0.23	1.94 ± 0.20	2.08 ± 0.21
60	0.37 ± 0.07	3.63 ± 0.47 **	3.71 ± 0.84 ^##^	16.70 ± 3.30	15.30 ± 5.32	13.50 ± 0.19	2.90 ± 0.07	3.10 ± 0.20	3.03 ± 0.41
90	0.46 ± 0.13	4.90 ± 0.85 **	4.63 ± 0.23 ^##^	26.10 ± 0.23	28.30 ± 2.12	26.30 ± 2.15	4.57 ± 0.29	4.19 ± 0.29	4.24 ± 0.26
120	0.88 ± 0.10	5.96 ± 0.38 **	5.65 ± 0.40 ^##^	46.00 ± 3.07	46.70 ± 1.50	47.30 ± 3.15	4.91 ± 0.05	4.91 ± 0.18	5.08 ± 0.32

**: GC vs. LQ, ** < 0.01; ^##^: JG-GC vs. GC, ^##^ < 0.01.

**Table 3:** Concentration of the analytes in JG–GC and GC.

Analyte	LQ	ILQ	LG	ILG	GLY
	(μg/mL)	(μg/mL)	(μg/mL)	(μg/mL)	(μg/mL)
JG–GC(3 g/mL)	7100	600	2000	70	26,000
GC(2 g/mL)	7500	560	2200	70	26,500

**Table 4:** Concentration of the analytes in each group.

Group	LQ (ng/mL)	LG (ng/mL)	GLY (ng/mL)
JG–GC (1.5 mg/mL)	3550	1000	13,000
GC (1 mg/mL)	3750	1500	13,250
LQ	3500	/	/
LG	/	900	/
GLY	/	/	13,200
LQ+JG	3500	/	/
LG+JG	/	900	/
GLY+JG	/	/	13,200

**Table 5:** Linear ranges and LLOQs of analytes in different biological matrixes.

Analyte	Rat Plasma		HBSS Buffer		Fecal Lysates	
	Linear Range	LLOQ	Linear Range	LLOQ	Linear Range	LLOQ
	(ng/mL)	(ng/mL)	(ng/mL)	(ng/mL)	(ng/mL)	(ng/mL)
LG	0.43–330	0.43	0.54–1100	0.54	2.15–2200	2.15
ILG	0.41–315	0.41				
LQ	0.47–360	0.47	1.47–3000	1.47	1.47–1500	1.47
ILQ	0.40–306	0.4				
GLY	10.26–7875	10.26	3.96–8100	3.96	5.27–5400	5.27
GA	14.64–11,250	14.64			2.93–3000	2.93
